# *ETV6::NTRK3* Fusion-Positive Wild-Type Gastrointestinal Stromal Tumor (GIST) with Abundant Lymphoid Infiltration (TILs and Tertiary Lymphoid Structures): A Report on a New Case with Therapeutic Implications and a Literature Review

**DOI:** 10.3390/ijms25073707

**Published:** 2024-03-26

**Authors:** Isidro Machado, Reyes Claramunt-Alonso, Javier Lavernia, Ignacio Romero, María Barrios, María José Safont, Nuria Santonja, Lara Navarro, José Antonio López-Guerrero, Antonio Llombart-Bosch

**Affiliations:** 1 Pathology Department, Instituto Valenciano de Oncología, Calle Gregorio Gea 31, 4to Piso, 46009 Valencia, Spain; 2 Patologika Laboratory, Hospital Quiron-Salud, 46010 Valencia, Spain; 3 Pathology Department, University of Valencia and CIBERONC, 46009 Valencia, Spain; antonio.llombart@uv.es; 4 Molecular Biology Unit, Instituto Valenciano de Oncología, 46009 Valencia, Spain; rclaramunt@fivo.org (R.C.-A.); jalopez@fivo.org (J.A.L.-G.); 5 Oncology Unit, Instituto Valenciano de Oncología, 46009 Valencia, Spain; jlavernia@fivo.org (J.L.); iromero@fivo.org (I.R.); 6 Radiology Department, Instituto Valenciano de Oncología, 46009 Valencia, Spain; mbarrios@fivo.org; 7 Oncology Unit, Hospital General de Valencia, University of Valencia and CIBERONC, 46009 Valencia, Spain; safont_mar@gva.es; 8 Pathology Department, Hospital General de Valencia, 46009 Valencia, Spain; nsantonja@uv.es (N.S.); lara.navarro@uv.es (L.N.)

**Keywords:** *GIST* wild-type, *ETV6::NTRK3* gene fusion, therapeutic implication, lymphoid infiltration

## Abstract

Gastrointestinal stromal tumors (GISTs) are the most common mesenchymal tumors of the gastrointestinal tract, with proto-oncogene, receptor tyrosine kinase (*c-kit*), or *PDGFRα* mutations detected in around 85% of cases. GISTs without *c-kit* or platelet-derived growth factor receptor alpha (*PDGFRα*) mutations are considered wild-type (WT), and their diverse molecular alterations and biological behaviors remain uncertain. They are usually not sensitive to tyrosine kinase inhibitors (TKIs). Recently, some molecular alterations, including neurotrophic tyrosine receptor kinase (*NTRK*) fusions, have been reported in very few cases of WT GISTs. This novel finding opens the window for the use of tropomyosin receptor kinase (TRK) inhibitor therapy in these subtypes of GIST. Herein, we report a new case of *NTRK*-fused WT high-risk GIST in a female patient with a large pelvic mass (large dimension of 20 cm). The tumor was removed, and the histopathology displayed spindle-predominant morphology with focal epithelioid areas, myxoid stromal tissue, and notable lymphoid infiltration with tertiary lymphoid structures. Ten mitoses were quantified in 50 high-power fields without nuclear pleomorphism. DOG1 showed strong and diffuse positivity, and CD117 showed moderate positivity. Succinate dehydrogenase subunit B (SDHB) was retained, Pan-TRK was focal positive (nuclear pattern), and the proliferation index Ki-67 was 7%. Next-generation sequencing (NGS) detected an *ETV6::NTRK3* fusion, and this finding was confirmed by fluorescence in situ hybridization (FISH), which showed *NTRK3* rearrangement. In addition, an *RB1* mutation was found by NGS. The follow-up CT scan revealed peritoneal nodules suggestive of peritoneal dissemination, and Entrectinib (a TRK inhibitor) was administered. After 3 months of follow-up, a new CT scan showed a complete response. Based on our results and the cases from the literature, GISTs with *NTRK* fusions are very uncommon so far; hence, further screening studies, including more WT GIST cases, may increase the possibility of finding additional cases. The present case may offer new insights into the potential introduction of TRK inhibitors as treatments for GISTs with *NTRK* fusions. Additionally, the presence of abundant lymphoid infiltration in the present case may prompt further research into immunotherapy as a possible additional therapeutic option.

## 1. Introduction

Gastrointestinal stromal tumors (GISTs) are the most frequent mesenchymal neoplasm of the gastrointestinal tract, and the majority occur in the stomach and the small intestine [1,2,3,4]. Receptor tyrosine kinase (*c-kit*) or platelet-derived growth factor receptor alpha (*PDGFRα*) mutations have been detected in around 85% of cases, and these cases without both mutations are considered wild-type (WT) GISTs [1,2,3,4,5,6,7]. WT GISTs are commonly not sensitive to tyrosine kinase inhibitors (TKIs) [7]. A small proportion of GISTs either reveal succinate dehydrogenase (SDH) deficiency or display murine sarcoma viral oncogene homolog B (*BRAF)* or *RAS* mutations [1,2,3,4,5,6,7]. The other GISTs without mutations in any of the previous genes have been classified as ‘quadruple WT’ GISTs [1,2,3,4,5,6,7], and additional molecular alteration, including the phosphatidylinositol-4,5-Bisphosphate 3-Kinase Catalytic Subunit Alpha (*PIK3CA)* mutation, fibroblast growth factor receptor 1 (*FGFR1*), *BRAF* or anaplastic lymphoma kinase *(ALK)* gene fusions, and neurotrophic tyrosine receptor kinase (*NTRK*) fusions (*ETV6::NTRK3* and *LMNA::NTRK1*), have been reported in quadruple WT GISTs [1,2,3,4,5,6,7].

The *NTRK* family consists of *NTRK1*, *NTRK2*, and *NTRK3*, which encode tropomyosin receptor kinase (TRK) [8,9]. *NTRK* fusion has been reported in many unrelated neoplasms, including infantile fibrosarcoma [10], mesoblastic nephroma [11], secretory breast carcinoma [12], mammary analog secretory carcinoma of the salivary gland [13], thyroid carcinoma [14], glioblastoma [15], cholangiocarcinoma [16], myofibroblast tumors [17], and WT GISTs [18,19,20,21,22,23]. Herein, we report a new case of quadruple WT with *ETV6::NTRK3* gene fusion, a rare genetic event with only a few cases previously reported. In addition, the present case displays an extensive lymphoid infiltration (tumor infiltrate lymphocytes as well as tertiary lymphoid structures) that offers new therapeutic options based on immunotherapy. Although *NTRK* fusions are rarely detected in WT GISTs at a low frequency, patients with GIST presenting *NTRK* fusions had a good chance of responding to treatment with TRK inhibitors, particularly patients with unresectable neoplasms, disseminate disease, or recurrent tumors that are resistant to TKIs. In such cases, TRK inhibitors, such as Larotrectinib and Entrectinib, may be a new therapeutic option [24,25,26,27,28,29,30]. Based on the present case, we further explored the clinicopathological and genetic features of previously reported GISTs with *ETV6::NTRK3* fusion.

## 2. Materials and Methods

### 2.1. Histopathology and Immunohistochemistry (IHC)

Histopathology was performed with the conventional hematoxylin and eosin method. IHC was performed on formalin-fixed, paraffin-embedded (FFPE) tissue sections with a thickness of 4 µm using an automated staining instrument (DAKO). Appropriated negative and positive controls were included. The testing and assessment were performed according to the manufacturer’s instructions for every biomarker. For Pan-TRK, nuclear, cytoplasmic, or membranous staining in more than 5% of tumor cells was considered positive [31,32,33,34]. All IHC-stained sections were interpreted blinded by two experienced pathologists (IM and ALLB).

### 2.2. Next-Generation Target Sequencing (DNA and RNA)

An Oncomine™ Comprehensive v3 panel (OCAv3) from Thermo Fisher Scientific (Based in Waltham, MA, USA) was used for sequencing. A list of the genes include in the panel is summarized in (https://assets.thermofisher.com/TFS-Assets/LSG/brochures/oncomine-comprehensive-assay-v3-flyer.pdf (accessed on 13 March 2024)).

Nucleic acids were extracted and quantified using the Genexus™Purification System with a Genexus™ FFPE DNA/RNA Purification Combo Kit (Thermo Fisher Scientific). Detection of genomic alterations was then performed using a Genexus™Integrated Sequencer. The OCAv3 is an amplicon-based, targeted assay that enables the detection of relevant single-nucleotide variants, amplifications, gene fusions, and indels from 161 unique genes. Genomic data were analyzed, and alterations were detected using the Ion Torrent Genexus Software 6.8.1.1. (Thermo Fisher Scientific). We also manually reviewed the variant call format file and integrated the Genomic Viewer. Only variants in coding regions, promoter regions, or splice variants were retained. 

The threshold for positive detection was defined at ≥25 RNA reads, originating from at least two unique PCR products, as determined by molecular barcoding. 

### 2.3. Fluorescence In Situ Hybridization

Formalin-fixed paraffin-embedded (FFPE) section tissue was used to perform fluorescence in situ hybridization (FISH). We utilized a commercial kit, the FISH Pretreatment kit (Vitro, Master Diagnostica^®^, Granada, Spain), to prepare the histological specimens according to a standardized protocol. This kit has been specifically designed for manual use to maintain a consistent procedure.

**Figure 1:**
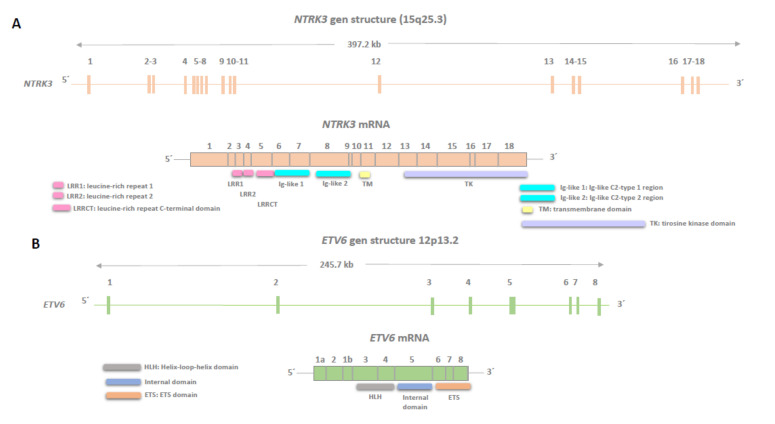
Genomic structures of neurotrophic tyrosine receptor kinase (*NTRK3*) (**A**) and ETS Variant Transcription Factor 6 (*ETV6)* (**B**) are shown, illustrating exons encoding the canonical isoforms as described in the Genome Browser v461 software. The regions of the corresponding mRNAs encoding functional domains are marked. *NTRK3* stands for Neurotrophic Tyrosine Kinase Receptor Type 3 (NCBI Gene ID: 4916), and *ETV6* stands for ETS Variant Transcription Factor 6 (NCBI Gene ID: 2120).

**Figure 2:**
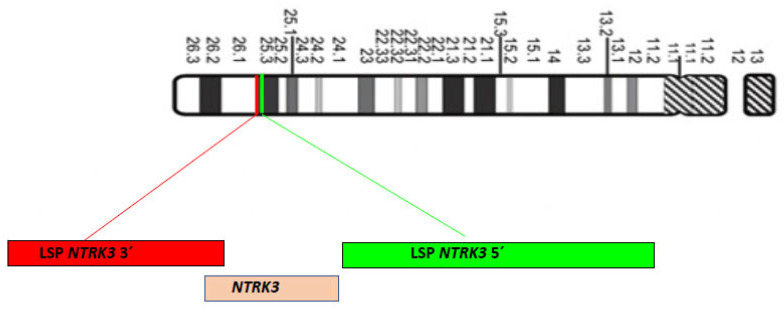
Chromosome 15 diagram, ISCN 2009, and localization of LSP neurotrophic tyrosine receptor kinase *NTRK3* 5′ (Green) and LSP *NTRK3* 3′ (Red) FISH probes on 15q25.3 chromosome position.

For the hybridization step, we employed the *NTRK3* Break Apart FISH Probe Kit by Cytotest^®^ (Rockville, MD, 208050, USA). This probe is tailored to detect rearrangements in the human *NTRK3* gene located on chromosome band 15q25.3. Apart from identifying breaks that may result in gene translocation, inversion, or fusion with other genes, the probe set can also detect other *NTRK3* aberrations, such as deletions or amplifications. The *NTRK3* Break Apart FISH Probe Kit spans approximately 500 Kb and covers the 5’ (start) portion of the ABL2 locus, and adjacent genomic sequences marked with CytoGreen fluorochrome (Figure 1 and Figure 2) show *NTRK* and *ETV* gene details and the probe design. The LSP *NTRK3* 3‘ FISH Probe spans approximately 720 Kb and covers sequences at the 3’ (end) of the gene marked with CytoOrange fluorochrome. These two probes flank sequences across the *NTRK3* locus where variable breakpoints have been observed. 

Following hybridization with the FISH probes, we cleaned the sections with a post-hybridization buffer to remove nonspecific labeling. Subsequently, we used a dapi-antifade mounting medium, included in the kit, to observe and preserve the fluorescence. The analysis was performed using a Zeiss Axio Imager fluorescence microscope with a 63X magnification. We counted 100 nuclei with the assistance of two independent observers (RC and JALG). *NTRK* rearrangements were interpreted based on the presence of a predominant atypical signal pattern with extra-signal *NTRK* 3′ in more than 10-15% of tumor cell nuclei and an isolated break-apart pattern [31,32,33,34].

## 3. Results

### Case Presentation

**Figure 3:**
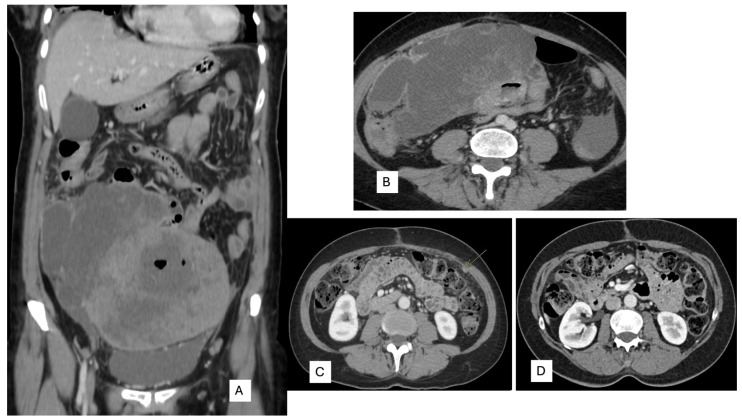
(**A**) Coronal and (**B**) axial. Computerized tomography displays a large intra-abdominal and pelvic tumor with necrosis attached to the small bowel. (**C**) Peritoneal carcinomatosis (arrow) in CT scan of follow-up. (**D**) CT scan after 3 months with Entrectinic treatment, showing a complete response.

**Figure 4:**
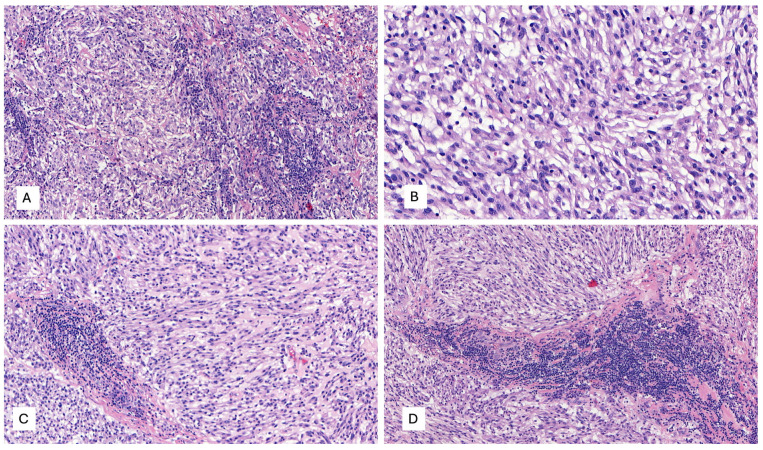
(**A**–**D**) Microscopic examination with hematoxylin and eosin (H&E) displays a mesenchymal neoplasm with spindle-predominant morphology, with focal epithelioid shape, ill-defined eosinophilic cytoplasm, myxoid stromal tissue, and remarkable lymphoid infiltration with tumor-infiltrating lymphocytes (TILs) and focal tertiary lymphoid structures, H&E. (**A**) 40×, (**B**) 400×, and (**C**,**D**) 200×.

A 53-year-old woman presented with complaints of abdominal and pelvic discomfort and was admitted to the hospital. The physician’s examination revealed a large intra-abdominal mass. Computerized tomography (CT scan) and magnetic resonance imaging (MRI) confirmed a large intra-abdominal and pelvic mass measuring 22 cm (Figure 3A,B). Surgical resection was performed, including hysterectomy, intestinal resection, and tumor removal. Although the surgeon performed an apparently complete margin-negative (R0) resection, the tumor was fragmented during the surgical procedure and could not be removed in a single specimen. Macroscopically, a large solid and cystic neoplasm measuring 30 cm. or larger dimension was observed, attached to the large and small bowel; the tumor displayed a gray-yellow-tan color and hard texture. Histopathology examination with hematoxylin and eosin displayed a mesenchymal neoplasm with spindle-predominant morphology, with focal epithelioid shape, ill-defined eosinophilic cytoplasms (Figure 4), and myxoid stromal tissue. Notable lymphoid infiltration with intratumoral and focal tertiary lymphoid structures was detected (Figure 4D). Ten mitoses were quantified in 50 high-power fields without nuclear pleomorphism.

**Figure 5:**
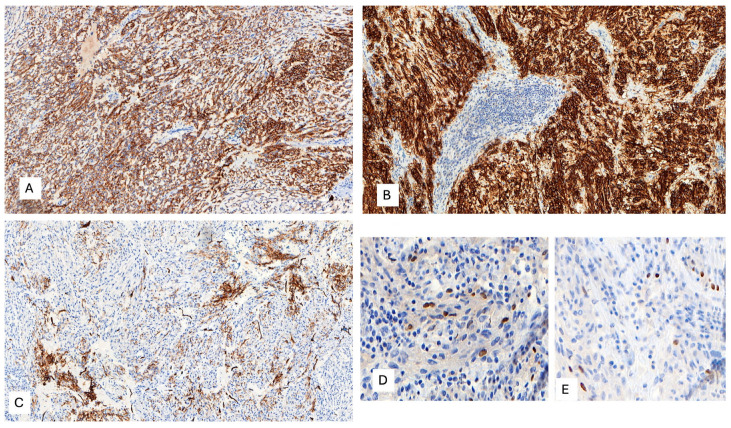
(**A**) Diffuse and moderate CD117 cytoplasmic immunoreactivity, 100×. (**B**) Strong and diffuse DOG1 cytoplasmic positivity, 200×. (**C**) Patchy CD34 positivity, 100×. (**D**,**E**) Focal and nuclear Pan-TRK immunoreactivity, 400×.

**Figure 6:**
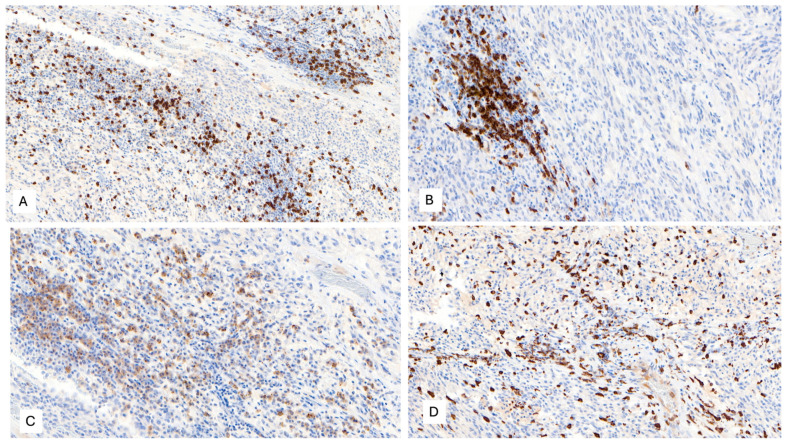
(**A**) CD8 positivity in tumor-infiltrating lymphocytes (TILs), 100×. (**B**) CD20 immunoreactivity in B-cells from tertiary lymphoid structures, 200×. (**C**) CD138 positivity in plasma cells, 100×. (**D**) CD163 positivity in the myeloid/histiocyte population, 100×.

**Figure 7:**
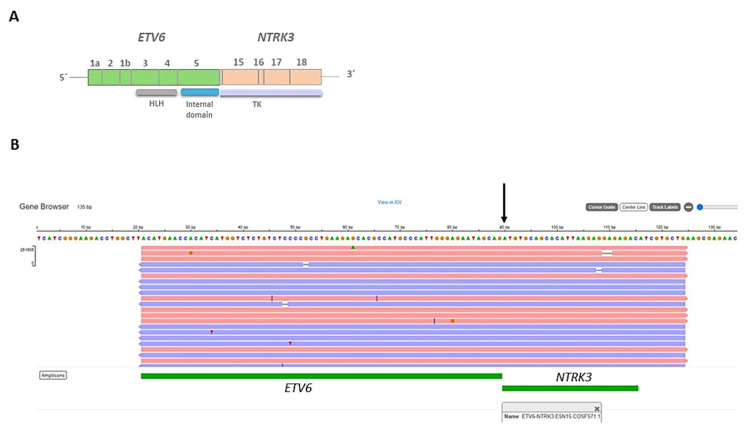
(**A**) Representation of the fusion gene (*ETV6::NTRK3*) obtained by next-generation sequencing (NGS), showing the exons and domains involved in the resultant fusion gene. HLH: Helix-loop-helix domain, TK: tyrosine kinase domain. (**B**) Integrative Genomics Viewer (IGV) displaying the *ETV6::NTRK3* fusion gene.

**Figure 8:**
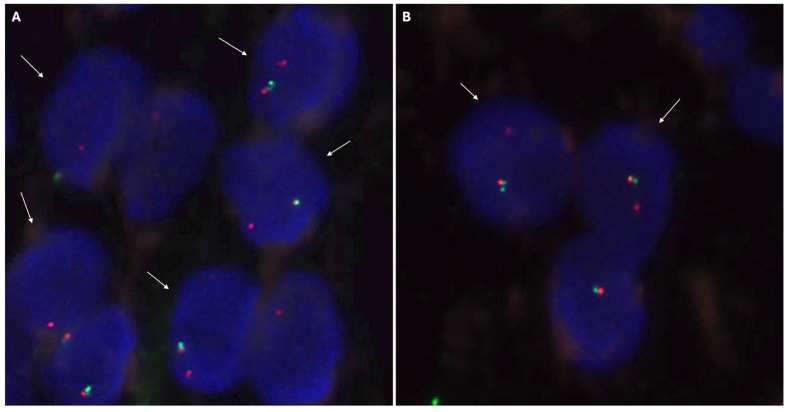
Neurotrophic tyrosine receptor kinase type 3(*NTRK3)* FISH analysis with a break-apart probe. (**A**) Positive nuclei for the rearrangement of the *NTRK3* gene, indicated by white arrows. The positive signal pattern corresponds to an atypical pattern with an extra *NTRK3* 3′ signal (red). Additionally, there are abnormal signal patterns with only one red signal and one normal nucleus with overlapping signals (*NTRK3* 5′ green and *NTRK3* 3′ red). (**B**) Two positive nuclei, indicated by white arrows, and one nucleus with a normal signal pattern. The positive nuclei present a typical positive signal pattern with separated *NTRK3* 3′ (red) and *NTRK3* 5′ (green) signals.

The IHC study revealed strong and diffuse DOG1 cytoplasmic positivity. CD117 showed moderate cytoplasmic immunoreactivity, and CD34 displayed focal positivity (Figure 5). SDHB was retained, and Pan-TRK showed focal and nuclear positivity (Figure 5D,E). The proliferation index Ki-67 was around 7%. S100, CK(AE1/AE3), Inhibin, estrogen receptors, calretinin, chromogranin, synaptophysin, smooth muscle actin, desmin, epithelial membrane antigen (EMA), CD31, anti-melanosome (HMB-45), and Melan A were all negative. CD3, CD4, and CD8 were positive in tumor infiltrate lymphocytes (TILs) (Figure 6A). CD20 highlighted the tertiary lymphoid structures (Figure 6B). CD138 was positive in plasma cells (Figure 6C). Programmed cell death 1 ligand (PDL1) displayed focal cytoplasmic/membranous positivity in tumor cells and PD-1 in lymphoid infiltration. CD163 showed diffuse positivity in the myeloid/histiocyte population (Figure 6D). Based on the histopathology and IHC results, the tumor was classified as a gastrointestinal stromal tumor (GIST) with a high risk of recurrence/metastasis. *ETV6::NTRK3* fusion was identified by RNA NGS (OCAv3 panel) and 251.517 reads (Figure 7), and *c-kit*, *PDGFRα*, *SDH*, *BRAF*, and *RAF* were all wild-type/non-mutated. In addition, we found *RB1* c.184C>T p.(Q62*), nonsense mutation, and an allelic frequency of 77.80%. An *NTRK3* rearrangement was detected by FISH (Figure 8). The lymphoid infiltration was found to be polyclonal both by immunohistochemistry and through polymerase chain reaction (PCR) and fragment analysis by Sanger sequencing.

A new CT scan performed one month after surgery revealed peritoneal carcinomatosis (Figure 3C), and Entrectinib was administered. After 12 weeks of drug intake, she presented with mild peripheral sensory neuropathy, non-severe pneumonia treated with antibiotics, mild constipation, and hypotension. A subsequent CT scan performed 3 months later showed a complete response (Figure 3D).

## 4. Discussion

Kinase-driven alterations, such as *NTRK* or *FGFR* fusion, have been recently reported in some GISTs [1,2,3,4,5,6,7,18,19,20,21,22,23]. The *NTRK* genes encode TRK proteins that exert oncogenic effects on tumors, and the activation of most TRK proteins is caused by *NTRK* fusions [8,9]. *NTRK* fusions occur in a variety of cancers with different incidences [8,9,10,11,12,13,14,15,16,17,18,19,20,21,22,23]. TRK inhibitors, such as Larotrectinib and Entrectinib, have shown encouraging antitumor efficacies in tumors with *NTRK* rearrangements and have been approved as treatments for multiple cancers harboring *NTRK* fusions [24,25,26,27,28,29,30]. Although the prevalence of *NTRK* fusions in GIST is extremely low and only a few patients were enrolled in the clinical trials, the antitumor efficacies of TRK inhibitors were observed [24,25,26,27,28,29,30]. Despite *NTRK* fusion being reported in some quadruple WT GISTs, only an *ETV6::NTRK3* fusion has been reported in eight cases, as presented in Table 1 [18,19,20,21,22], including the present case.

The clinicopathological features of GISTs with *ETV6::NTRK3* fusion are variable, and many of them have been reported in the fourth or fifth decade of life, predominantly in intestinal and pelvic locations, with variable dimensions but usually presenting as tumors with high dimensions [18,19,20,21,22]. They may exhibit spindle or epithelioid morphology and typically have a high risk of recurrence and metastasis [18,19,20,21,22]. The majority display DOG1 and CD117 immunohistochemical expression, with SDH retained and variable Pan-TRK immunoreactivity, often nuclear and focal [18,19,20,21,22]. FISH analysis has confirmed *NTRK3* rearrangement in five out of eight cases [18,19,20,21,22]. In localized disease, the usual approach is surgical treatment, but in metastatic and/or recurrent settings, several drugs have been employed, including Sunitinib, Sorafenib, Nilotinib, Imatinib, Larotrectinib, and Entrectinib [18,19,20,21,22]. The last two drugs seem to be very effective in tumors with *NTRK* rearrangement. The behavior depends on several factors, such as initial metastatic disease, tumor size, location, etc., but in the previously mentioned GISTs with *ETV6::NTRK3*, six patients were alive with or without the disease, and two died from the disease, with a median follow-up of 43 months [18,19,20,21,22].

Although the prevalence of *NTRK* fusions in GIST is extremely low and only a few patients were enrolled in the clinical trials, the antitumor efficacies of TRK inhibitors were observed [24,25,26,27,28,29,30]. In clinical trials (LOXO-TRK-14001, SCOUT, and NAVIGATE), 55 patients with tumors carrying *NTRK* rearrangements, including three GISTs, were enrolled to evaluate the efficacy of Larotrectinib [24,25,26,27,28,29,30]. All three patients with GIST experienced tumor shrinkage >30%, and one had a pathological complete response with sufficient tumor shrinkage >90% [24,25,26,27,28,29,30]. Furthermore, Larotrectinib has been recommended by the Belgian multidisciplinary expert panel as a first-line treatment for WT GIST with *NTRK* fusions [30]. These pieces of evidence [24,25,26,27,28,29,30], as well as our results, suggest that *NTRK* fusions define a unique subgroup of GIST, and TRK inhibitors have the potential to benefit GISTs with *NTRK* fusions. Hence, it is clinically significant to screen for *NTRK* fusions in WT GIST.

One challenging issue is determining the best method for screening *NTRK* fusion in WT GIST [31,32,33,34]. FISH has been recommended as the gold standard for detecting gene rearrangements, including break-apart probes and fusion probes; however, it does not provide information about partner genes involved in the fusions [31,32,33,34]. As *ETV6* is the most frequent partner in *NTRK3* fusions, we presume that many of the previously reported GISTs with *NTRK3* rearrangement harbor *ETV6* as a partner, as reported by Castillon et al. [20]. Although FISH appears more suitable for verification than for screening gene fusions in tumors with a low incidence of the fusions, NGS may provide information on broad-spectrum molecular alterations in many genes, including mutations, amplifications/deletions, gene fusions, and SNPs [31,32,33,34,35,36]. However, NGS requires strict quality control and may not be available in all hospitals. Compared to FISH and NGS, IHC is a cheap and fast testing method and is available in many hospitals. IHC staining for Pan-TRK has been used to screen *NTRK* fusions in several cancers [31,32,33,34,35,36,37]. However, both the sensitivity (50%) and specificity (16.7%) of Pan-TRK staining are low [22], and the stain may be focal, as observed in our study. Therefore, the validity of Pan-TRK IHC staining in screening *NTRK* rearrangements is uncertain in GISTs. The detection of a specific fusion partner in *NTRK* rearrangement tumors would not be mandatory in cases where positive *NTRK* FISH results involve the tyrosine kinase domain. Additionally, targeted therapy may be administered independently of the *NTRK*-specific subtype (1, 2, or 3) and the fusion gene partner.

The present case also reveals an *RB1* mutation, which seems to be associated with high-risk/malignant GISTs [38]. Our case was histologically classified as high-risk/malignant GIST.

Regarding the differential diagnosis, the relationship between mesenchymal tumors of the gastrointestinal tract with *NTRK* rearrangement and GIST is interesting [33]. Likely, the morphological differences are not quite evident, but the absence of CD117 and DOG1 immunohistochemical expression permits the distinction of *NTRK*-rearranged tumors from GISTs and highlights important phenotypic differences between these tumor types [33]. In fact, gastrointestinal mesenchymal tumors (CD117 and DOG1 negative) with *NTRK* rearrangements demonstrate substantial clinical and morphological heterogeneity and are generally unrelated to GIST [33]. 

In the era of precision medicine, with the agnostic approval of Entrectinib and Larotrectinib for metastatic tumors with *NTRK* fusions, we consider either of these two drugs to be the treatment of choice for patients with metastatic GIST and *NTRK* fusion, as in this case. The extremely low frequency of GISTs with *NTRK* fusions means that we have little evidence of the benefit of *NTRK* inhibitors in these tumors, making it especially important to collect real-world data to confirm the long-term benefit of this treatment in this patient group. One limitation of the present study is the short period of follow-up for our patient treated with Entrectinib. However, despite a peritoneal lesion described in the first follow-up CT scan, a new CT scan conducted 3 months later revealed a complete response, providing evidence of the effectiveness of Entrectinib treatment.

Finally, an unreported histopathological and IHC finding in our case but not in previous GISTs with *ETV6::NTRK3*, to the best of our knowledge, was the prominent TILs and plasmacytic infiltration, as well as tertiary lymphoid structures throughout the tumor. These features would open the possibility of a new treatment approach for this patient with immunotherapy; however, these findings should be confirmed in additional GISTs with *ETV6:NTRK3* gene fusion and GISTs or mesenchymal neoplasms of the gastrointestinal tract with *NTRK* rearrangement. This research should be conducted through multicenter studies with a large sample size. Nevertheless, these findings could be potentially used to guide personalized treatments for patients with GIST.

Future studies may further build on the genetic profile of so-called quadruple WT GISTs, which are likely not truly WT, and establish links between genomic kinase drivers (*NTRK* and *FGFR1* rearrangement) and therapeutic regimens.

## Figures and Tables

**Table 1:** Clinicopathological features of GIST WT with *NTRK3::ETV6* gene fusion.

Case Reference	Age/Gender	Location/Size	Morphology	Mitoses/ 50 HPF	Risk Classification	Lymphoid Infiltration	IHC Profile	NGS	FISH	Treatment	Progression/ Months	Status/Months
Brenca et al., 2016 [18]	44/M	Rectum/5 cm	Epithelioid	34	high	ND	DOG1+++,CD117:++ CD34:+, SDHB retained PanTRK: ND	*NTRK3::ETV6*	*ETV6* split	Surgery	No/44	Alive/44
Shietal 2016 [19]	55/M	Small bowel/NA	NA	NA	NA	ND	SDHB retained	*NTRK3::ETV6*	ND	Sunitinib Sorafenib Nilotinib Regorafenib Larotrectinib Imatinib	Yes/NA *	AWD/159
Shietal 2016 [19]	54/M	Colon/NA	NA	NA	NA	ND	SDHB retained	*NTRK3::ETV6*	ND	Sunitinib Sorafenib Linsitinib	Yes/NA **	AWD/12
Castillon et al., 2021 [20]	59/M	NA	NA	NA	NA	ND	DOG1+++,CD117:++ SDHB:ND PanTRK: negative	*NTRK3::ETV6*	*NTRK3 positive*	No	No/O	DOD/O
Lee et al., 2022 [21]	43/M	Rectum/11 cm	NA	0	high	ND	DOG1:ND,CD117:++ SDHB:ND PanTRK: +, weak	*NTRK3::ETV6*	*NTRK3 positive*	Surgery	No/36	Alive/36
Cao et al., 2023 [22]	52/F	Pelvic/13.3 cm	Spindle	8	high	ND	DOG1+++,CD117:++ CD34:+ SDHB: retained PanTRK:+	*NTRK3::ETV6*	*NTRK3 positive*	Surgery	Yes/11	DOD/11
Cao et al., 2023 [22]	56/M	Stomach/16 cm	Epithelioid	3	Intermediate	ND	DOG1+++,CD117:++ CD34:+, SDHB: retained PanTRK: negative	*NTRK3::ETV6*	*NTRK3 positive*	Surgery Imatinib	No/58	Alive/58
Present case	57/F	Pelvic/27 cm	Spindle	20	high	TILs and lymphoid nodules	DOG1+++.CD117:++ CD34:+, SDHB: retained PanTRK:+ focal	*NTRK3::ETV6*	*NTRK3 positive*	Surgery and Entrectinib	Yes/1	Alive/3

Abbreviations: M: male; F: female; ND: not described; NA: not available. * The patient experienced tumor progression during treatment with five lines of TKI therapy (155 months) and then achieved an ongoing partial response (44%) after Larotrectinib therapy for 4 months. ** The patient experienced tumor progression during treatment with four lines of TKI therapy (12 months). AWD: alive with disease, DOD: death of disease. Split: break-apart present. (Modified from Cao et al., 2023) [22].

## Data Availability

The datasets used and analyzed during the current study are available from the corresponding author upon reasonable request.
